# Evaluation of cytotoxic T lymphocyte-mediated anticancer response against tumor interstitium-simulating physical barriers

**DOI:** 10.1038/s41598-020-70694-8

**Published:** 2020-08-12

**Authors:** Shu-Ching Chen, Po-Cheng Wu, Chiao-Yi Wang, Po-Ling Kuo

**Affiliations:** 1 Department of Medical Research, National Taiwan University Hospital, Taipei, 10002 Taiwan; 2 Graduate Institute of Biomedical Electronics and Bioinformatics, National Taiwan University, Taipei, 10617 Taiwan; 3 Department of Electrical Engineering, National Taiwan University, Taipei, 10617 Taiwan; 4 Department of Physical Medicine and Rehabilitation, National Taiwan University Hospital, Taipei, 10002 Taiwan

**Keywords:** Biotechnology, Applied immunology, Cancer, Cancer microenvironment, Cancer therapy, Tumour immunology, Motility, Cancer, Cancer microenvironment, Cancer therapy, Tumour immunology

## Abstract

Tumor antigen-specific cytotoxic T lymphocyte (CTL) is a promising agent for cancer therapy. Most solid tumors are characterized by increased interstitial fluid pressure (IFP) and dense collagen capsule, which form physical barriers to impede cancer treatment. However, it remains unclear how CTL-mediated anticancer response is affected at the presence of these obstacles. Using a microfluidic-based platform mimicking these obstacles, we investigated the migration characteristics and performance of anticancer response of CTLs targeting hepatic cancer cells via antigen-specific and allogeneic recognition. The device consisted of slit channels mimicking the narrow interstitial paths constrained by the fibrous capsule and increased IFP was simulated by applying hydrostatic pressure to the tumor center. We found that antigen-specificity of CTLs against the targeted cancer cells determined the cytotoxic efficacy of the CTLs but did not significantly affect the success rate in CTLs that attempted to infiltrate into the tumor center. When increased IFP was present in the tumor center, CTL recruitment to tumor peripheries was promoted but success of infiltration was hindered. Our results highlight the importance of incorporating the physical characteristics of tumor interstitum into the development of CTL-based cancer immunotherapy.

## Introduction

Tumor antigen-specific CD8^+^ cytotoxic T lymphocyte (CTL)-mediated killing of tumor cells has a crucial role in cancer immunotherapy^1^. Success of CTL-mediated tumor rejection requires the recruitment, infiltration, and expansion of tumor antigen-specific CTLs in tumor interstitium—the fluidic and matrix compartments between vessels and tumor cells, and recognition and killing of the tumor cells by the CTLs^2^. However, a large body of evidence indicates that tumor cells actively reprogram surrounding interstitium to restrict CTLs from interacting with the tumor cells^3^. For example, many types of cancer upregulate endothelins signalling of tumor endothelium to impede CTLs infiltration in tumor^4,5^; soluble mediators such as IL-10 and transforming growth factor β (TGF-β) secreted by either tumor cells or tumor-recruited Treg cells significantly suppress the cytotoxic function of CTLs^3^. While a multitude of chemical factors employed by cancers to escape from anticancer immunity are disclosed^6^, an increasing interest has recently been gained in the physical barriers established by tumors in their interstitium, which also poses a significant challenge to CTLs to successfully contact the targeting cells^7,8^.

Direct delivery of immune cells into tumor interior via perfusion may be physically hindered by the increased vascular resistance imposed by the high compressive stress generated by tumor growth^9,10^. The growth-induced solid stress is mainly contributed by the collagen network and space-taking molecules, such as hyaluronan, accumulated in the tumor interstitium^11^. Strategies to improve the delivery of blood-borne therapeutic agents against tumor, including the anticancer immune cells, has emerged based on decompression of the tumor vessels by depletion of the collagen or hyaluronan, or increase of the flow rate of tumor vessels by normalizing the immature phenotype of the vascular network^8,10^. For example, improvement of tumor perfusion and consequently the efficacy of chemotherapy by stress alleviation and vascular normalization in solid tumors has been shown in vivo using losartan^12^, tranilast^13^, dexamethasone^14^, pirfenidone^15^, vismodegib^16^, metformin^17^, enzymes degrading collagen or hyaluronan^15,18,19^, and antiangiogenic agents for vascular normalization, such as bevacizumab^20^, an antibody against vascular endothelial growth factor (VEGF), and cediranib^21^, an inhibitor of VEGF receptor tyrosine kinase. In particular, scheduling lower-dose application of antibody against VEGF receptor 2 has been shown to enhance the infiltration of CTLs in breast tumor^22^. Losartan is a clinically approved antihypertensive drug that blocks angiotensin receptor and downregulates collagen and hyaluronan levels in tumor interstitium by inhibiting the fibrotic signaling pathway^12^. Tranilast is a clinically approved anti-allergic drug but also effective in suppression of collagen synthesis partially via inhibition of TGF-β_1_^13,23^. Dexamethasone, a glucocorticoid steroid widely used in a variety of diseases, inhibits hyaluronan expression in tumor and normalize tumor vessel phenotype by blocking angiogenesis signaling^14^. Pirfenidone downregulates collagen production in fibroblast mainly via inhibition of TGF-β_1_ signaling and is clinically approved for treatment of idiopathic pulmonary fibrosis^24^. Vismodegib is clinically approved for treatment of basal cell carcinoma and lessens the proliferative activity of cancer-associated fibroblasts as well as the expression of collagen and hyaluronan in tumor interstitium mainly via inhibition of sonic-hedgehog pathway^16^. Metformin, a widely used anti-diabetic drug, inhibits TGF-β_1_ signaling and reduces the production of collagen and hyaluronan in tumor^17^.

When the perfusion into tumor interior is compromised, therapeutic agents, including infiltrating CTLs, are anticipated to accumulate primarily in the tumor peripheries^18,25^. Two physical obstacles typically encountered by the CTLs managing to infiltrate in the tumor peripheries are dense collagenous layers and high interstitial fluid pressure (IFP)^7,26^. In most solid tumors, tumor islets are surrounded by layers of condensed fibrillar network that is mainly composed of collagen and fibronectin and the network is more condensed in regions adjacent to the islet boundary than that in area far away from the islet^7^. The narrow spacing between the fibrils restricts T cells from contacting tumor cells and those networks with fibril spacing smaller than 5 μm are nearly void of T cells^7^. Together with the abundant water-trapping molecules such as hyaluronan, the condensed interstitial matrix compromises the interstitial hydraulic conductivity and contributes to the increase of IFP in tumors^8,10,26^. Compared with normally negative IFP, most solid tumors have elevated IFP ranging from 500 to 8,000 Pa^27–30^. The IFP is almost a constant throughout the tumor center and drops precipitously at the tumor boundaries^31^. The increased IFP and the steep pressure gradient contribute to a reduced transcapillary transport of therapeutic agents in tumor and may impede the migration of CTLs through the tumor interstitium^32^. The elevated IFP further promotes the motility and invasiveness of the cancer cells^33^. The elevation of IFP frequently coincides with the high solid stress in most solid tumors and strategies targeting stress alleviation in tumor are also effective in IFP reduction^13,15,16,18,19^. However, the dynamics and efficacy of CTL-mediated killing of cancer cells at the presence of these two obstacles has been barely addressed. Platforms that evaluate the efficacy of CTL-mediated tumor rejection in an environment recapitulating these two physical barriers, are thus indispensable to the improvement and optimization of cancer vaccine strategy.

In this paper, we investigated the behaviors of GP33 antigen-specific CTLs against GP33+/− murine hepatic cancer cells HEPA1-6 at the presence of elevated IFP and narrowed interstitial paths that were simulated by a platform based on microfluidics, which has recently gained interest in studies of interactions between cancer and immune cells^34–39^. Migration characteristics of the CTLs in the simulated interstitial paths, with and without the presence of increased IFP, were studied. Performance of the CTL-mediated killing of the cancer cells was quantitatively evaluated by calculating the number of CTLs that managed to migrate through the thin path, the time spent by the CTL to move through the thin path, the percentage of CTLs successfully transmigrating the path, the number of cancer cells killed by a single CTL, and the time spent by individual CTLs to kill a cancer cell. To address the importance of antigen-recognition specificity, the performance profile of the GP33-antigen specific CTLs against HEPA1-6.GP33+ cells were compared with that of 2C T-cell receptor (TCR) transgenic CTLs against hepatic cancer cells BNL, which were recognized by the T cells via not antigen-specific, allogeneic recognition.

## Results

### The microfluidic platform

**Figure 1:**
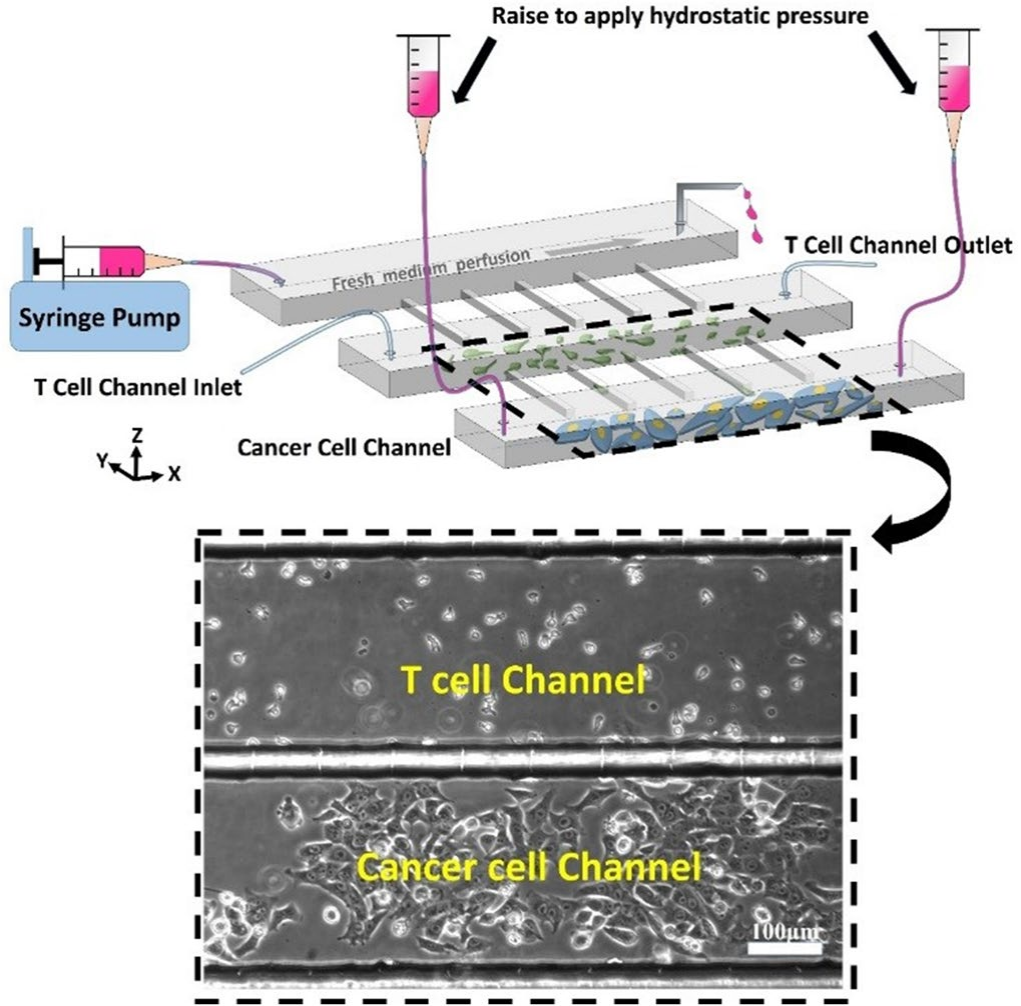
Schematic and photographic representation of the microfluidic platform simulating physical obstacles in tumor interstitium, which consisted of three 50 μm high main channels interconnected by 5 μm high slit channels. Fresh medium was supplied via the top channel at a flow rate of 0.1 μL/min. CTLs and tumor cells were cultured in the middle and bottom channel respectively, which were referred to as T-cell and cancer-cell channel for clarity. Elevated IFP was simulated by rising the medium-containing syringes that were connected with the cancer-cell channel above the platform. Scale bar: 100 μm.

A schematic of the microfluidic platform is shown in Fig. 1. The microfluidic platform consisted of three main channels running in parallel and an array of “slit” channels connecting adjacent main channels. The main channels were 50 μm high, 300 μm wide, 8 mm long, and equally separated by 30 μm. The slit channels were located at the floor of the main channels and had a width of 2 μm and a height of 5 μm, which were smaller than the average size of T cells (7–10 μm) and only one T cell was allowed to pass through at a time. The platform was designed to simulate a simplified tumor microenvironment with the three main channels representing a tumor center, an adjacent interstitial space full of recruited CTLs, and a feeding vessel respectively. Thus for clarity, the top, middle, and bottom main channels shown in Fig. 1 were referred to as vessel, T-cell, and cancer-cell channel respectively. The side walls between adjacent main channels represented the physical barriers surrounding the feeding vessel and the tumor center and the slit channels were designed to mimic the narrow fenestration in vessel wall and tumor capsule. Increased IFP in the tumor center was simulated by imposing hydrostatic pressure (HP) to the bottom channel by elevating syringes that were full of medium and connected with the channel above the platform.

### CTL-mediated killing of tumor cells

There were two pairs of murine hepatic cancer cells and CTLs employed in this work. The first pair consisted of BNL cancer cells and 2C CD8^+^ T cells that had L^d^-allogeneic recognition against the BNL cells. The second pair were composed of GP33+/− HEPA1-6 cancer cells as well as GP33-antigen specific P14 CD8^+^ T cells. To facilitate cell adhesion and migration inside the microfluidic platform, the T-cell and cancer-cell channels of the platform were first incubated with a solution of 5 μg/mL intercellular adhesion molecule 1 (ICAM-1, R&D System) and 25 μg/mL fibronectin (Sigma-Aldrich) for 1 h at 37 °C, respectively, followed by flushing with phosphate buffer saline (PBS). About 600 cancer cells were then seeded into the cancer-cell channel and incubated for 5 h at 37 °C and 5% CO_2_ to allow the cells to attach to the glass and spread. After that, about 5 × 10^4^ CTLs were seeded into the T-cell channel and time-lapse live cell images were acquired to trace the behaviors of individual cells.

**Figure 2:**
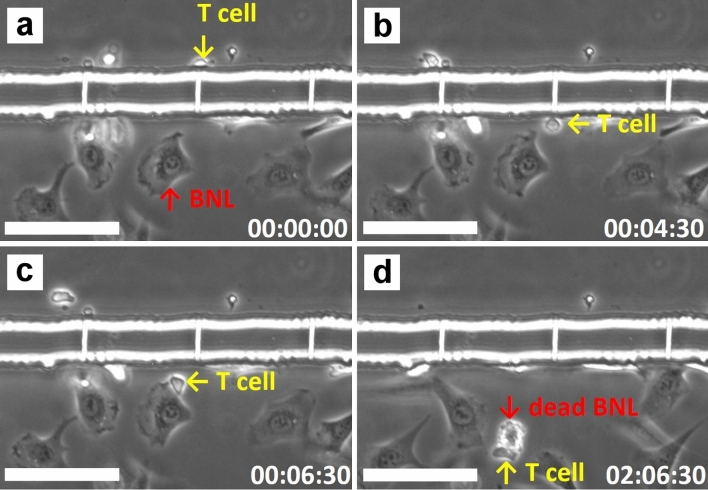
Tumor-interstitium infiltration and killing of a BNL cell executed by a 2C CD8^+^ T cell. The T cell and targeted BNL cell were denoted by a yellow and red arrow, respectively. (**a**) The T cell moved to the opening of slit channel. (**b**) The T-cell successfully transmigrated through the slit channel and moved to cancer-cell channel. (**c**) The T cell attached to the targeted BNL cell, and (**d**) apoptosis of the targeted cell occurred roughly two hours thereafter. The scale bars are 100 μm, and time is expressed as hh:mm:ss.

Figure 2 demonstrates an example of CTL-mediated killing of tumor cells in the tumor interstitium-mimicking platform without applying elevated HP to the cancer-cell channel. A 2C CD8^+^ T cell was seen to migrate to the opening of a slit channel (Fig. 2a), manage to transmigrate through the channel (Fig. 2b), attach to a BNL cell (Fig. 2c), and bring about apoptosis of the tumor cell, which was characterized by rapid blebbing and subsequent shrinkage and fragmentation of the cell (Fig. 2d; see also Movie M1). Since the average size of T cells was slightly larger than the opening of the slit channels, it took about 4.5 min for the T cell to deform and squeeze its cell body through the slit channel. The time that the T cell spent to kill the BNL cell was about 2 h. Supplementary Figure S1 and Movie M2 exhibit examples of the anti-GP33+ HEPA1-6 cancer cells response mediated by the GP33-antigen specific P14 CTLs. Successful transmigration of CTL into the cancer cell channel was first seen at time 2:06:00. Of great interest is the CTL (annotated by the yellow arrow) that managed to transmigrate through the slit channel at time 3:58:30. It successfully moved to the cancer cell channel at time 4:04:30 but immediately moved away from the cancer cell positioned close to the slit through which the CTL transmigrated (from time 4:10:00 to 4:21:30). However, the CTL wandered back to the cancer cell about 17 min later (time 4:27:00), and attached to the cancer cell at time 4:43:30. Another CTL denoted by the red arrow transmigrated through the same slit channel at time 4:17:00 and behaved similarly to the yellow arrow-annotated one. Apoptosis of the cancer cell finally occurred around time 6:45:00. These results highlight the chemotactic nature in the initiation of the CTL-mediated anticancer response.

**Figure 3:**
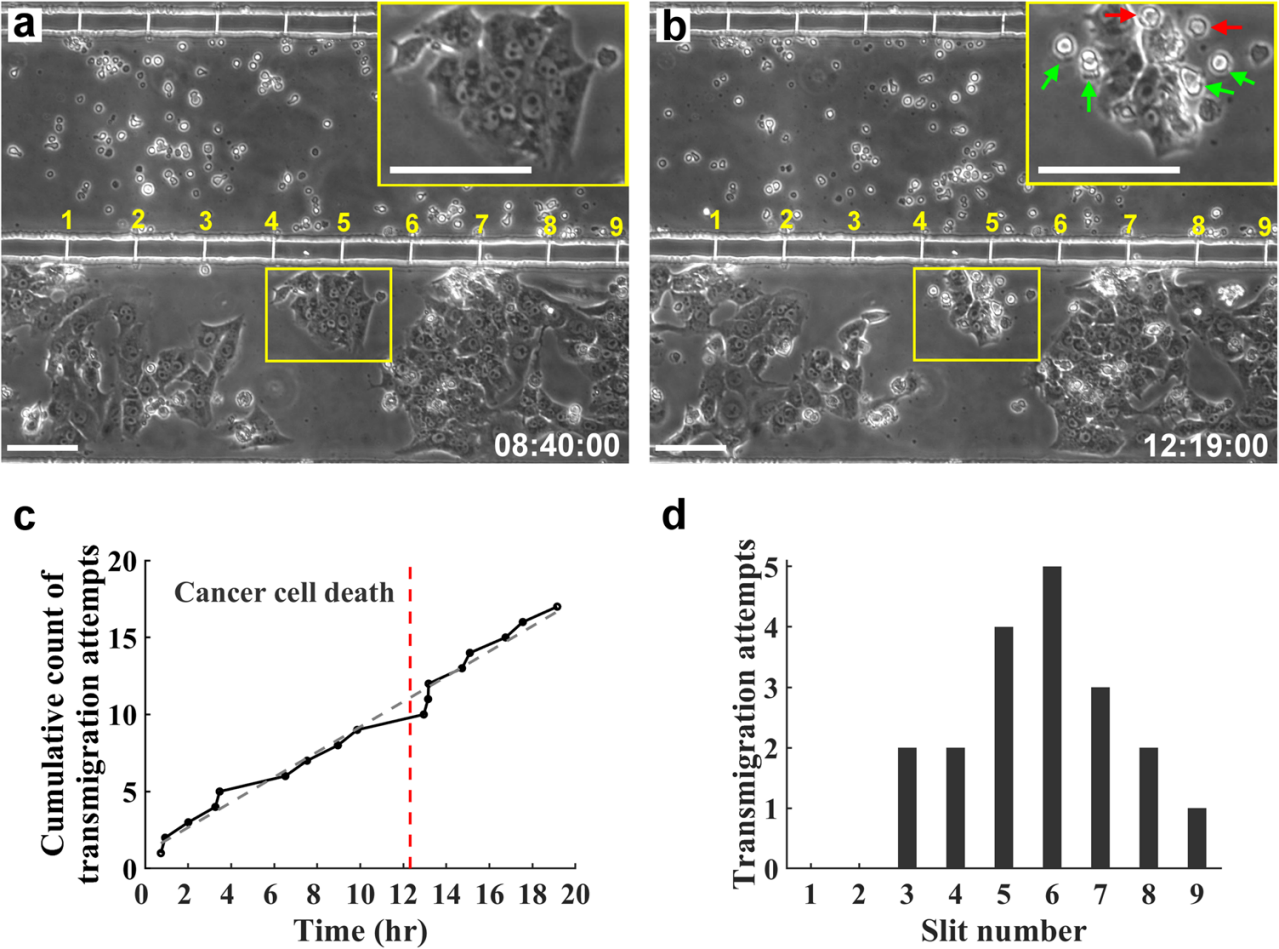
T-cell recruitment enhanced by apoptosis of cancer cells. Phase contrast images of cultured T cells and HEPA1-6.GP33+ cancer cells acquired (**a**) before and (**b**) after apoptosis of the cancer cells located near and in between the slit channel #5 and #6. The cell cluster was marked by the yellow box. Inserts: magnified images of the cell cluster with the apoptotic cells close to the slit channel highlighted by red arrows. Green arrows denote other apoptotic cells in the cluster. Scale bars: 100 μm. Time is expressed as hh:mm:ss. (**c**) The temporal profile of the cumulative count of the incident when a CTL sought to transmigrate through a slit channel to the cancer-cell channel. The dashed line represents the linear regression of the cumulative counts against time. The local slope of the profile is interpreted as the trend of the CTLs managing to transmigrate at the moment, and the slope of the linear regression is considered as the rate of transmigration owing to background migration. The vertical dash-line denoted when apoptosis of cancer cells denoted by the red arrow in (**b**) occurred. (**d**) Histogram of the incidents of transmigration attempt that occurred at individual slit channels throughout the experiment. Channel numbers are defined in (**a**) and (**b**).

A critical step in CTL-mediated anticancer response is the recruitment of tumor antigen-specific T cells at places presenting dense soluble tumor antigens, which are largely released from dead tumor cells. Figure 3 shows an example of CTL recruitment enhanced by death of tumor cells. The T-cell and cancer-cell channel were cultured with GP33-targeting CD8^+^ T cells and HEPA1-6.GP33+ cells, respectively, and there was no elevated HP applied to the cancer-cell channel. To facilitate data comparison, the slit channels shown in Fig. 3a,b were numbered from left to right. In Fig. 3a, there was initially a cluster of HEPA1-6 cells located near slit channel #5. At 739 min after image recording, apoptosis of a tumor cell very close to the opening of the slit channel, as annotated by the red arrow shown in the inset in Fig. 3b, was seen in this cluster. Note that there were at least 5 other apoptotic tumor cells seen in the cluster, as highlighted by green arrows in the inset of Fig. 3b.

Figure 3c depicts the cumulative count of the incident when a CTL moved to the opening of a slit channel and sought to transmigrate through the channel. We defined the incident of transmigration attempt as that a CTL managed to squeeze through a slit channel by protruding a long cell process into the channel, whether the cell successfully moved to the cancer-cell channel or not. The cumulative counts were profiled against time and superimposed with the linear regression of the data represented by the dashed line. The local slope of the profile is interpreted as the trend of the CTLs managing to transmigrate at the moment, and the slope of the linear regression is considered as the rate of transmigration owing to background migration. While most of the local slopes are close to the background one, apparently the rate of transmigration attempt increased immediately after the apoptosis of the HEPA1-6 cell shown in Fig. 3b, which occurred on the time denoted by a vertical dashed line in Fig. 3c. This suggests that the CTL recruitment may be transiently enhanced after the cancer cell death. Analysis of the slit channels where transmigration attempts occurred throughout the experiment further revealed that most transmigration attempts occurred at slit channels close to the dead tumor cell (i.e., #5 and #6), as shown in Fig. 3d. Together, these data demonstrate the capacity of our platform to investigate the dynamic of CTL recruitment, infiltration, and killing of tumor cells in a tumor interstitium-mimicking environment.

### Performance of CTL-mediated anticancer response

**Figure 4:**
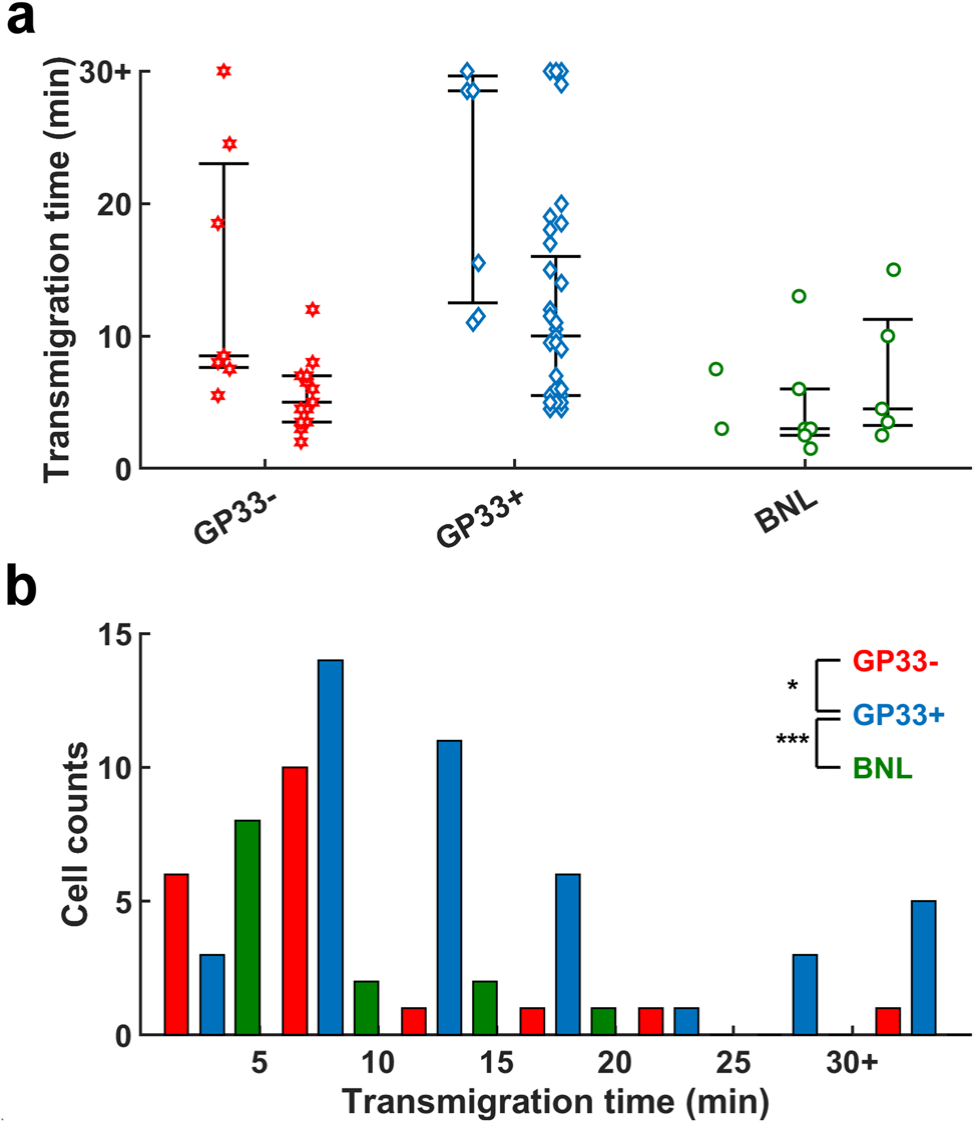
Distribution of the successful transmigration time of individual CTLs. (**a**) Scatter plots of the time spent by individual CTLs to transmigrate through the slit channels when the T-cell and cancer-cell channels were cultured with P14 and HEPA1-6.GP33-, P14 and HEAP1-6.GP33+, and 2C and BNL cells, respectively. Data were collected from 2, 2, and 3 individual experiments for the GP33+, GP33−, and BNL cancer cells, respectively. (**b**) Histogram of the transmigration time when data of the same condition were pooled together. * and *** indicate *p* < 0.05 and *p* < 0.001 respectively.

**Figure 5:**
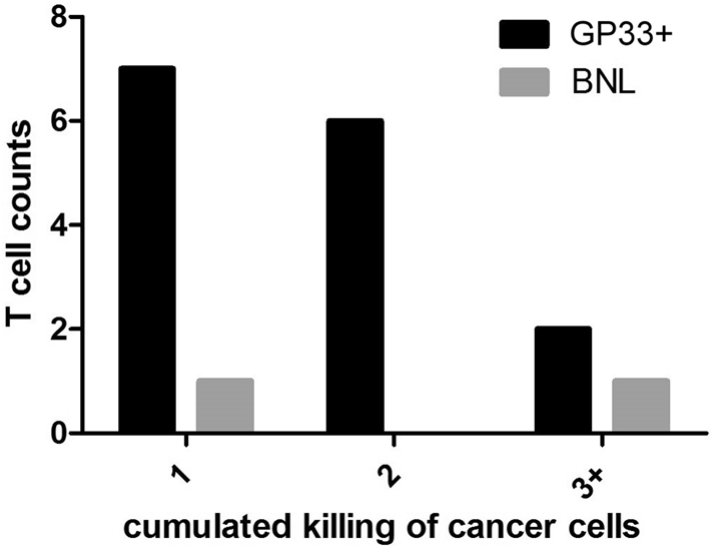
Number of HEPA1-6.GP33+ cells killed by individual P14 CTLs and that of BNL cells killed by individual 2C CTLs.

The presented in vitro platform allows quantitative measurement of the efficacy of CTL-mediated anticancer response in a tumor interstitium-mimicking environment. Specifically, we evaluated the performance of CTLs attacking cancer cells via antigen-specific and allogeneic recognition by measuring the success rate that individual CTLs attempted to transmigrate through a slit channel, the time spent for individual CTLs to transmigrate through the slit channels, the rate of CTLs that successfully transmigrated into cancer-cell channel and killed cancer cells, the number of cancer cells killed by individual CTLs, the time that individual CTLs spent to kill a cancer cell, and summarized the results in Figs. 4, 5, and Table 1. Note that these data were acquired in conditions without application of elevated HP to the cancer-cell channel. The data were collected from 2, 2, and 3 independent experiments in conditions with that the T-cell and cancer-cell channels were cultured with P14 CTLs and HEPA1-6.GP33+, P14 and HEPA1-6.GP33-, and 2C and BNL cells, respectively.

**Table 1:** Transmigration characteristics of CTLs toward different kinds of cancer cells (P14 against HEPA1-6.GP33+ and HEPA1-6.GP33−, and 2C against BNL); data presented in the first two rows are expressed as mean(range); and data presented in the third row are expressed as median (range).

Cancer cell type	HEPA1-6. GP33+	HEPA1-6. GP33−	BNL
Transmigration attempts	40(19 − 62)	35(30 − 41)	9(7 − 10)
Success rate of transmigration	0.47(0.36 − 0.58)	0.3(0.17 − 0.43)	0.5(0.2 − 0.7)
Transmigration time (min)	10.5(4.5 − 99.5)	6.7(2 − 32.5)	3.5(1.5 − 13)
Killing ratio	36%	N/A	15%
Killing time (min)	194.4 ± 125.5	N/A	80.5 ± 33.9

Many solid tumors are surrounded by dense collagenous capsule. Success of CTLs to transmigrate through the dense capsule is a prerequisite for an effective CTL-based anticancer immune response. In the present work, the PDMS barrier between the T-cell and cancer-cell channel represented a 30 μm thick dense capsule with 2 × 5 μm thin paths. Since the data shown in Fig. 3 suggest that the chemicals released by dead tumor cells had a role in attracting CTLs, we asked whether the success of transmigration of CTLs were affected by the specificity of the CTL against the antigen expressed by the tumor cells. The success rate of transmigration is defined as the number of CTLs that managed to transmigrate the slit channels dividing that of CTLs that successfully moved into the cancer-cell channel.

As summarized in Table 1, we found that compared with 2C CTLs, there were more P14 CTLs attempting to transmigrate through the thin slits in individual experiments. The averaged success rates of transmigration were close in conditions when the cultured CTL and cancer cell pairs were P14 versus HEPA1-6.GP33+, and 2C versus BNL, respectively, although there is large variation between individual experiments (37%, 58% for CP33+, and 20%, 60%, and 71% for BNL, respectively). In contrast, the averaged success rate was smaller when the cell pairs were P14 versus HEPA1-6.GP33-(17% and 43% in individual experiment). These data implicate the role of tumor-antigen specificity in the infiltration of CTL in the tumor surrounding. However, the considerable variation between individual experiments implies the stochastic nature of the success transmigration. Note that there were totally 50 slit channels between the T-cell and cancer-cell channels and the number of transmigration attempt could be underestimated since we only observed 9 slit channels in individual experiments. Indeed, we did find T cells that wandered into the cancer-cell channel from the leftmost or rightmost margin of the channel on the acquired image, which suggests that there were transmigrations of CTLs in slit channels outside of the image. Nevertheless, we believe that the data were representative since the constraints of image acquisition were the same for the three types of cancer cells.

Figure 4 depicts the time spent for individual CTLs to transmigrate through a slit channel in various conditions. The transmigration time did not vary significantly across the individual experiments in each condition (Fig. 4a). When the data were pooled together, the transmigration time exhibited a Poisson distribution, with that > 80% CTLs took less than 20 min to move through the channel (Fig. 4b). Statistical analysis revealed that the transmigration times of P14 CTLs against HEPA1-6.GP33+ were significantly longer than those of P14 CTLs against HEPA1-6.GP33-(*p* < 0.05) and 2C CTLs against BNL (*p* < 0.001), respectively. This finding suggests that the P14 versus HEPA1-6.GP33+ condition may draw CTLs to dedicate more time to overcome the physical barrier before giving up. The median of the estimated transmigration speeds were 2.8, 4.4, and 6 μm/min for P14 CTLs against HEPA1-6.GP33+, HEPA1-6.GP33-, and for 2C CTLs against BNL cells, respectively. Note the estimated transmigration speeds were far slower than the migratory speeds of CTLs measured in the T-cell channel (~ 10 μm/min). This is reasonable since it took more time for the CTLs to squeeze through the tight, narrow slits.

After successfully transmigrating from the T-cell channel into the cancer-cell channel, some CTLs were seen to rove between and over cancer cells, while some adhered to individual cancer cells for a while and eventually brought about apoptosis of the cells. We found that during progress of the cancer-cell apoptosis, the CTLs moved across rather than staying still on the surface of the targeted cells. Among the forty-two P14 CTLs transmigrating into the HEPA1-6.GP33+ culturing channel, fifteen CTLs were found to successfully induce cancer-cell apoptosis. In contrast, among the thirteen 2C CTLs that successfully moved into the BNL culturing channel, there were only two CTLs performing cancer-cell killing throughout the experiment. The cancer-cell killing rates—defined as the ratio of number of CTLs executing cancer-cell killing to that of CTLs successfully transmigrating to the cancer-cell channel—were about 36% and 15% when the targeted cells were HEPA1-6.GP33+ and BNL, respectively (Table 1). However, the number of the CTLs that killed the BNL cells was too small and additional studies are required to determine whether antigen-specific recognition is more effective than allogeneic recognition in CTL-mediated killing of cancer cells.

Since not all CTLs that successfully transmigrated into the cancer-cell channel conducted cancer-cell killing, of great importance to the success of CTL-mediated anticancer response is the number of cancer cells eradicated by individual CTLs. We found that many CTLs killed more than one cancer cell throughout the experiment. As shown in Fig. 5, there were over 50% P14 CTLs killed more than one encountered HEPA1-6.GP33+ cells; in one experiment, we even saw that 5 HEPA1-6 cells were sequentially killed by one CTL. There were totally 27 HEPA1-6 cells killed by 15 CTLs and one CTL killed 1.8 cancer cells in average. In comparison to the results in the cumulated killing of HEPA1-6 cells, there were totally 5 BNL cells killed by 2 2C CTLs and the average killing rate was 2.5 cancer cells per CTL. Note that the comparison could be biased due to the small number of 2C CTLs that were observed to kill the cancer cells.

An important parameter regarding the performance of CTL-mediated anticancer response is the time that individual CTLs spend to kill a cancer cell. It is expected the shorter the time spent for cancer killing, the better the anticancer immune response. As shown in Table 1, it took the P14 CTLs roughly 200 min to kill a HEPA1-6.GP33+ cell, and 80 min for 2C CTLs to kill a BNL cell. The time spent for individual CTLs to kill a cancer cell in the present work was longer than that recently reported by Ritter et al., which was roughly 20 min^40^. Such a discrepancy could result from the fact that the targeted cells used by Ritter et al. were lymphoma cells, which were much smaller than the hepatic cancer cells used in the present work.

### Effects of elevated HP on CTL-mediated anticancer response

**Figure 6:**
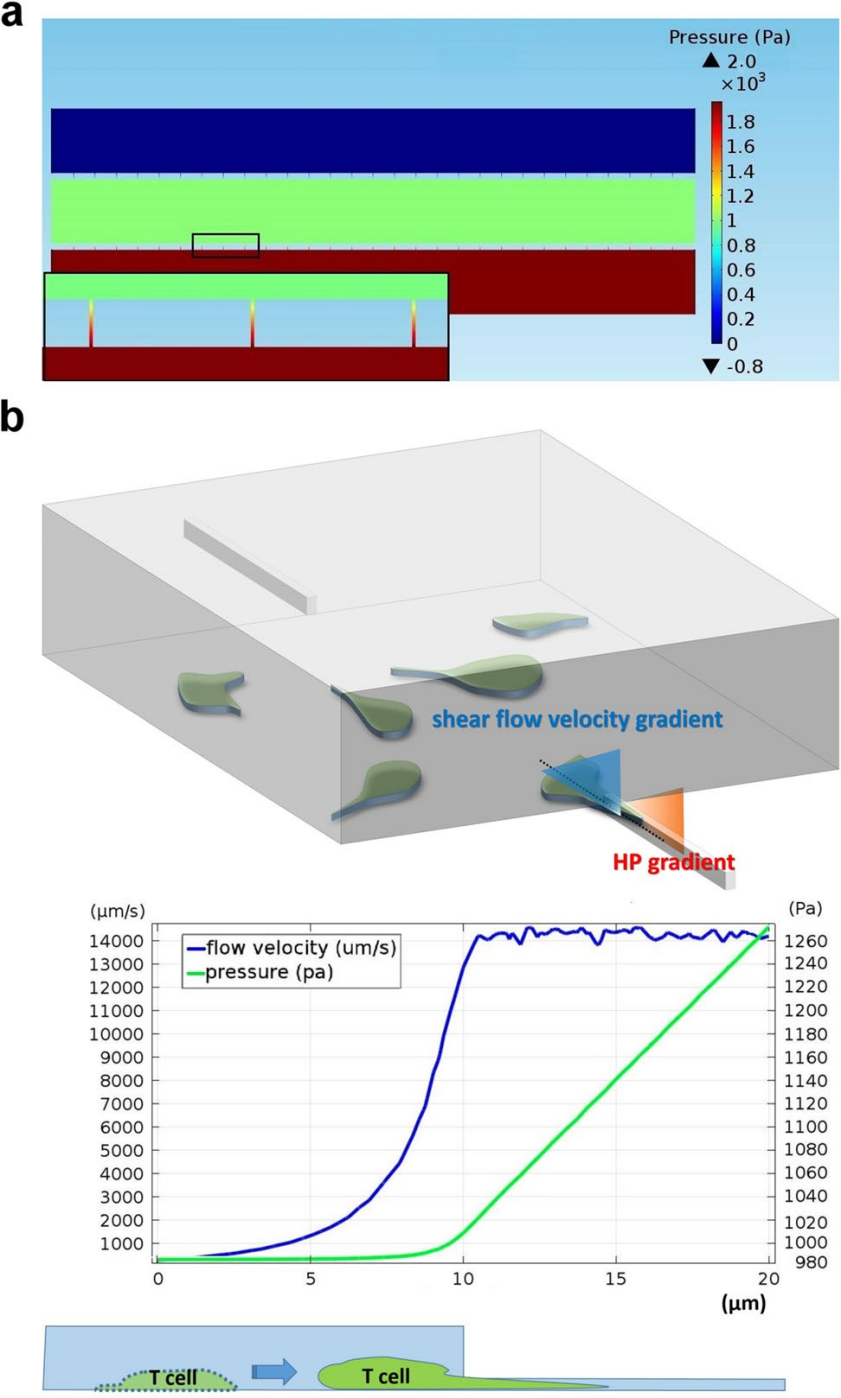
Simulated hydrodynamics profile in the tumor-interstitium-mimicking device. (**a**) Computed pressure distribution inside the device when a HP of 20 cmH_2_O (~ 1960 Pa) was imposed to the cancer-cell channel and a continuous flow rate of 0.1 μL/min was set in the vessel channel. The pressure distributions inside the T-cell and cancer-cell channels were generally homogeneous and an abrupt pressure drop was seen in the slit channels, which were magnified in the inset for clarity. (**b**) Schematic of T-cell transmigration at the peripheries of the simulated tumor center and the spatial profiles of flow velocity (blue) and HP (green) along a slit channel. The HP gradient inside the channel propelled a continuous flow through the channel at a rate about 14 mm/s, which dropped abruptly at the opening of the slit channel toward the T-cell channel. Note that the slit channel was 30 µm long and only the profile data along one third of the channel length were plotted for clarity.

The high IFP conditions frequently seen in solid tumours were simulated by elevating the syringes connected to the inlet and outlet of the cancer-cell channel to the same heights above the device. Figure 6a demonstrates computation results of pressure distribution within the device when a HP of 20 cmH_2_O (~ 1960 Pa) was applied to the cancer-cell channel. The computed pressure in the T-cell channel was approximately half of that in the cancer-cell channel. The pressure exhibited a homogeneous distribution inside both channels and there was an abrupt pressure drop in the slit channels connecting the two channels, which was consistent with those observed in vivo^31^. The HP gradient inside the channel drove a continuous flow through the channel at a rate about 14 mm/s, which dropped abruptly at the channel opening toward the T-cell channel, as shown in Fig. 6b. Measuring the pressure in individual channels using a pressure gauge further confirmed that when a HP of 20 cmH_2_O was applied to the cancer-cell channel, the measured pressures in the T-cell and cancer-cell channel were 1,853 and 1,040 Pa, respectively, which were close to the computed results. The slight disparity between the computed and experimental results may result from the errors in setting the syringes and device connection.

To examine the effects of increased HP in tumor center on the performance of CTL targeting the tumor cells, we applied HPs of 5, 10, and 20 cm H_2_O—approximately corresponding to 490, 980, and 1960 Pa—to the cancer-cell channel cultured with HEPA1-6.GP33+ cells and tracked the motion of P14 CTLs. When the cancer-cell channel was pressurized with a HP about 1960 Pa, the estimated amount of fluid flowing from the cancer-cell channel to the T-cell channel throughout the experiment was about 15 μL/h, which resulted in a negligible drop (~ 2 mm for an 8 h experiment) of fluid level in the elevated syringes.

**Figure 7:**
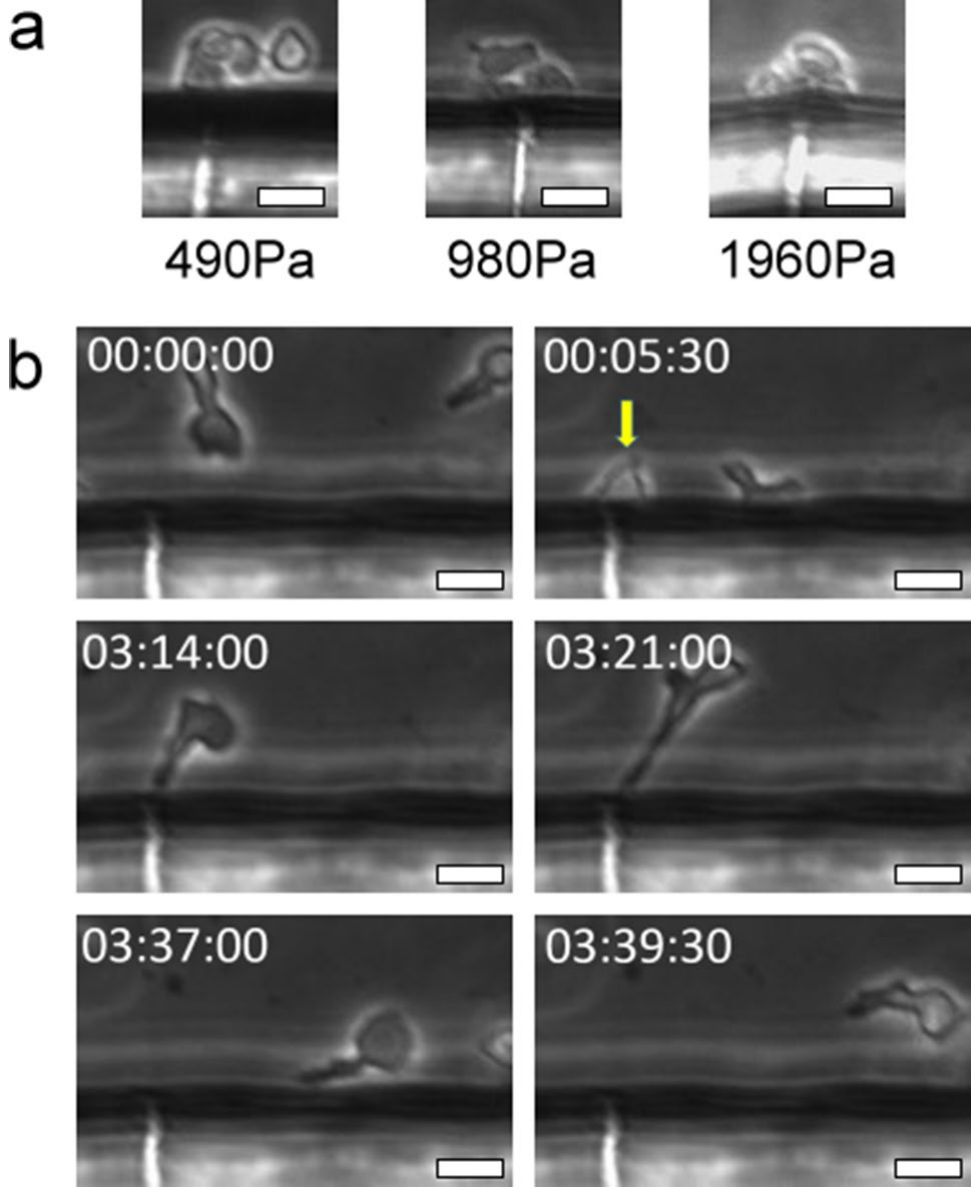
Morphological characteristics of CTLs close to slit channels when the cancer-cell channel was pressurized. (**a**) CTLs formed a crowd near the opening of slit channels when various HPs were applied to the cancer-cell channel. (**b**) The “lollipop”-like appearance of CTLs after the cells failing to transmigrate and leaving the opening of a slit channel.

We found that application of HPs in the cancer-cell channel did impede the transmigration of the CTLs; the numbers of CTL successfully transmigrating into the cancer-cell channel were 1, 0, and 0 when the applied HP was 490, 980, and 1960 Pa, respectively, throughout the 8 h experiment. However, the CTLs did attempt to approach the cancer cells as there were frequently crowds of CTLs seen at the opening of the slit channels (Fig. 7a). To elaborate this phenomenon, we used COMSOL to simulate the flow condition inside the slit channel when HP was applied to the cancer-cell channel. We found that there was a shear flow of high velocity (~ 14 mm/s) near the opening of the slit channel toward the T-cell channel (Fig. 7b). T cells are known to crawl upstream against the direction of flow when the substrate surface was coated with ICAM-1^41,42^. Thus, when there was a pressure gap between the cancer-cell and T-cell channel, the resulting shear flow near the opening of the slit channel could draw the CTLs to move to the vicinity of the opening and to try to migrate against the flow and squeeze themselves through the slits. This was supported by the observation that when a CTL failed to transmigrate through the slits and moved backward, it usually exhibited a “lollipop”-like shape with a long sprout (Fig. 7b, see also Movie M3). The long sprout could last for a couple of min and was seldom seen in experiments when there was no HP applied to the cancer-cell channel.

## Discussion

In the present work, we reported a microfluidic-based platform and evaluated the performance of the CTL-mediated cancer cell rejection at the presence of physical barriers simulating tumor interstitium. Many methods based on radioactivities, enzyme-linked immunospot, and flow cytometry have been developed to evaluate the efficacy of CTL-mediated tumor rejection both in vitro and in vivo. In these methods, anticancer immune responses are quantitatively determined from information either associated with lysis of target cells, or related to engagement of pathways specific for cytotoxicity^43–45^. However, none of these approaches allows investigation of CTL-mediated tumor rejection in contexts mimicking the physical constraints in tumor interstitium, which is characterized by narrow entrances confined by dense collagenous capsule and high IFP that both physically impede CTL infiltration. Our findings indicate that the presence of the physical barriers has prominent effect on the performance of the CTL-mediated anticancer response.

The simulated narrow entrances and high IFP condition blocked over half and all of the patrolling CTLs managing to approach the tumor center, respectively. Interestingly, it appears that the chance of the CTLs successfully transmigrating through the simulated narrow entrances is not dependent on the presence and types of tumor-antigen specificity. This finding implies that the type of the CTL-recognition against the cancer cells does not affect the ability of the CTLs to squeeze themselves across the thin paths. However, the presence of the tumor-antigen-specific recognition may allow a larger and more heterogeneous population of CTLs to be attracted to the tumor peripheries, while in the non-specific and allogeneic-recognition conditions, the CTLs that attempted to transmigrate the slit channels were probably composed of a small population of CTLs that were highly motile and active in exploring environment. This may explain why it took longer time for the P14 CTLs to transmigrate through the thin paths when the cells were co-cultured with the GP33-expressing cancer cells. But further studies are required to delineate these issues. Nevertheless, our data suggest that the antigen-specificity between the CTLs and targeting cancer cells appears to affect the recruitment of the CTLs to the tumor surrounding, as evaluated by the number of transmigration attempt, but does not influence the success ratio of the CTLs to infiltrate through the tight tumor interstitium. Likewise, our data indicate that the HP gradient at the peripheries of a tumor center imposes an obstacle to prevent from successful infiltration of CTLs to attack the cancer cells. In a three-dimensional context like our slit condition, T cells are known to propel themselves forward by generating an intracellular HP gradient^46^, and this propelling process is expected to be hindered if the pressure gradient inside the slit channel exceeds the intracellular one.

There are several limitations in our approach. Our cells were cultured in 2D conditions and may behave differently from those cultured in 3D spaces mimicking the in vivo conditions. Pavesi et al. investigated the efficacy of CTLs against tumor cells in dispersion or aggregates using a microfluidic device with three long channels running in parallel and separated by trapezoidal posts^37^. Type I collagen gel containing hepatocellular carcinoma cells (HCC) expressing hepatitis B virus (HBV) antigen was synthesized in the middle channel, while the side channels were flowed with CTLs engineered to express specific T cell receptors (TCR) recognizing the HBV antigen. They found that in the 3D condition, hypoxia hindered the infiltration of the engineered CTLs into the cancer cell-populated collagen gel and consequently reduced cancer killing, which was not observed in their 2D model. Using a similar platform, Lee et al. determined the effect of monocytes, a major component in tumor stroma, on the killing efficacy of CTLs engineered against HCC expressing HBV antigen^36^. They found that in the 3D microfluidic condition, the presence of monocytes significantly suppressed the anticancer response of CTLs expressing HBV-specific TCR prepared by retroviral transduction, while the monocyte-mediated suppression did not occur when the HBV-specific TCR CTLs were produced by mRNA electroporation. By contrast, such a discrepancy was not seen in their 2D model. Olofsson et al. recently reported their work studying the interaction between natural killer (NK) cells, the granular lymphocytes lacking TCR, with the human leukaemia K562 cells, when both were co-cultured in collagen gels confined in an array of microwells^47^. They found the NK cells exhibited large heterogeneity in motility in the confined space. Parlato et al. developed a microfluidic platform and cultured interferon-α (INF-α) conditioned dendritic cells (DCs), the very cells capturing and presenting tumor antigens to lymphocytes, in its central channel flanked by two side channels containing collagen gel embedded with colorectal cancer cells^38^. The channels were interconnected with thin microgrooves. They found the motion trajectory of the DCs in the 3D space was driven toward the cancers treated with an epigenetic drug romidepsin and INF-α (RI), but not toward those without treatment, and the RI treatment-driven DC migration was blocked by CXCR4 inhibition. However, the stiffness of the collagen gels synthesized in these studies is usually too soft to fully recapitulate the mechanical characteristics of the dense fibrous capsule surrounding a solid tumor^48^.

Another drawback in the present work is that the quantification demanded tons of human labor and limited the throughput. There are a multitude of high throughput, 2D platforms reported for studies of the interaction between immune cells and tumor cells at single cell level. The most popular approaches were to passively confine the interacting cells using an array of microwells, hydrodynamic traps, or picoliter droplets, such that the cell–cell interactions could be precisely defined in space and time, and multiplexed measurement was allowed. For example, Yamanaka et al. co-deposited the K562 cells with NK cells in microwells and investigated the cytolytic and secretory behaviors of the latter at the same time^49^. They found that the secretion of INF-γ from the NK cells was independent of the NK cell-mediated cytolysis, which was most probable during the first encountering of an NK cell with the tumor cell. The Voldman’s group has published a series of works regarding pairing cells using hydrodynamic traps, which allowed the deterministic control of the initiation of cell–cell interactions, including CTL and the antigen-presenting cells^50,51^. Sarkar et al. probed the cytolytic activities of individual NK cells co-encapsulated with lymphoma cells in picoliter droplets generated by a microfluidic device^52^ and demonstrated that a higher efficiency of the derivative NK cell line NK-92 in eradication of primary B-cell non-Hodgkin lymphoma cells than the primary NK cells. Christakou et al. examined the variation of one hundred NK-mediated cytolysis against major histocompatibility complex class (MHC) I-deficient tumor cells in parallel by utilizing ultrasound to synchronize the contacts between the two types of cells in a multiwell chip^53^. The Fan’s group interrogated the paracrine signaling between thousands of paired microphage and glioma cell by co-culturing the cells in an array of microwells attached to a poly-L-lysine functionalized glass slide that was patterned by a high-density antibody barcode array using a flow patterning technique^54^. They confirmed the existence of glioma-macrophage communication at the single cell level by showing that the profile of protein expressed by the co-cultured cells was different from the addition of that of purely macrophage and tumor cells. Abonnenc et al. reported a platform patterned with grid electrodes and forced the contact between CTLs and lymphoblastoid cells by entrapping the cells in dielectrophoresis cages and routing the paired cages via the electrical network^55^. While most of the aforementioned platforms were based on optical approach for data acquisition, Charwat et al. developed a sophisticated platform to study the dynamics of CTLs-mediated anticancer response by utilizing impedance sensing and optical light scattering to quantify the amount of the adherent tumor cells and that of the nonadherent CTLs, respectively^39^. However, none of these platforms addressed the physical barriers imposed in the present work, nor was the ability of the immune cells to overcome these barriers considered when the efficacy of the immune cell-mediated antitumor response was evaluated. The last major limitation in the present work is that the molecular basis governing the migratory characteristics of the CTLs exposed to the pressure barrier was not addressed. Hence, further improvements including mechanistic investigation are required in our system.

In summary, we developed a microfluidic-based platform to quantitatively evaluate the performance of CTL-mediated anticancer response at the presence of physical barriers commonly seen in tumor interstitium, including the narrow interstitial paths and elevated IFP. Migration characteristics of CTLs against these barriers was analyzed. Performance of cancer cell rejection was evaluated and compared between cancer cells that were targeted by CTLs via antigen-specific and allogeneic recognition. We found that the presence of the physical barriers did affect the performance of CTL-mediated anticancer response. Our results highlight the importance of incorporating the physical characteristics of tumor interstitium in evaluation of the efficacy of CTL-mediated tumor rejection, and are anticipated to expand our capacity in the development and optimization of cancer vaccine strategies.

## Methods

### Devices fabrication

The device was fabricated using a two-step soft lithographic approach and replicate molding^56,57^. A 2 μm thick SU-8 2002 photoresist (MicroChem Corp., Newtown, MA) film was first spin-coated on a silicon wafer and exposed to UV with a photomask to pattern the slit channels, which consisted of 50 parallel-aligned, 2 μm wide, and 1,200 μm long lines that were equally separated by 100 μm. After development, a second 50 μm thick SU-8 2050 layer was spin-coated on top of the first layer and exposed to UV using a photomask with layout configuration of the three main channels. The yielded master was silanized and poured with a degassed mixture of polydimethylsiloxane (PDMS, Sylgard 184, Dow Corning) pre-polymer and curing agent at 10:1 w/w ratio, followed by baking for 4 h at 65 °C. The cured PDMS replica was peeled off from the master, punched at the inlet and outlet of the main channels using a biopsy punch (Miltex, USA), and bonded to a glass slide after oxygen plasma treatment using an oxygen plasma cleaner (PDC32G, Harrick Plasma, USA). The glass slide formed the floor of the microfluidic channels and provided a surface for subsequent cell adhesion.

### Cell culture

The cancer cells BNL and HEPA1-6 were obtained from American Type Culture Collection (ATCC) and maintained in Dulbecco’s modified Eagle’s medium, supplemented with 10% fetal bovine serum (FBS), 1% penicillin/streptomycin (P/S) and 0.1% 2-mercaptoethanol (2-ME) at 37 °C and 5% CO_2_. GP33 expression was promoted by adding G418 into the culture medium for HEPA1-6 cells. 2C and P14 CD8^+^ T cells were harvested from 2C and P14 TCR-transgenic mice, respectively, as previously described^58^. The 2C and P14 TCR-transgenic mice were kindly provided by Dr. John T. Kung in Academia Sinica, Taipei, Taiwan. The 2C transgenic mice carry functional rearranged TCR α-(one copy) and β-(eight copies) chain transgenes from a cytotoxic T-cell clone 2C specific for L^d^ MHC class I antigen. The P14 transgenic mice possess rearranged TCR transgenes specific for the gp33 epitope (amino acid 33–41 of glycoprotein) of lymphocytic choriomeningitis virus (LCMV). All animals were kept in a specific pathogen-free facility and were used at age of 4–6 weeks. The CD8+ T cells with purity above 99% were obtained from spleens of mice by positive isolation of CD8+ T cells and sorted by FACSAria (BD Bioscience, San Jose, CA, USA). The P14 CD8+ specific for LCMV gp33 in the context of H-2Db were generated by activating the P14 naive CD8+ T cells with 10 μg/ml mitomycin C-treated LPS-activated syngeneic B cell blasts and M2 peptide of 0.08 nM for 3 days. The activated T cells were washed and cultured in the presence of human recombinant IL-2 (rhIL-2, 100 IU/mL) for 2–3 days. The 2C CD8+ effector T cells were generated by activation of 2C naïve CD8+ T cells by QL9 peptide. Cells were sgrown at 37 °C and 5% CO_2_ in humidified air. All procedures for animal management were performed following the guideline of the Use of Laboratory Animals published by National Taiwan University and approved by Institutional Animal Care and Use Committee of College of Medicine and College of Public Health of National Taiwan University.

### Imaging acquisition and data analysis

After the seeded cancer cells adhered and spread well in the cancer-cell channel, the device was mounted to a motorized stage on a phase-contrast microscope (IX71, Olympus, Japan) equipped with an environmental chamber that was kept at 37 °C and 5% CO_2_ during image acquisition. About 5 × 10^4^ CTLs were seeded into the T-cell channel. Fresh medium was pumped into the top main channel using a syringe pump (Fusion 200, CHEMYX, USA) at a flow rate of 0.1 μL/min and mass transport was allowed between adjacent main channels by simple diffusion via the slit channels. Time-lapse live cell images were taken every 30 s using a 10X objective with numerical aperture of 0.25 and a CCD camera (pixelfly, Pco. Germany). The positions of individual CTLs were determined by their centroids that were manually tracked from the acquired images using the Image J software.

### Statistical analysis

The normality of data distribution was examined by the Lilliefors test. For normally distributed data, the significance of differences between means were evaluated by student *t*-test or ANOVA for grouped data with Tukey’s post hoc test. For non-normally distributed data, the Kruskal–Wallis and multiple comparison test were employed. A *p*-value less than 0.05 was considered to be significant.

### Pressure application and COMSOL simulation

HP was applied to the cancer-cell channel by elevating the syringes connected to the inlet and outlet of the channel to the same heights above the device. The syringes elevation varied from 5 to 20 cm, which were equivalent to 490–1960 Pa. The plungers of the syringes were removed and the conduit from the syringe to the cancer-cell channel was full of medium. After the syringes were fixed at a predetermined height above the device, the syringe rear was covered with parafilm to prevent from medium evaporation during cell culture. The pressure in the T-cell and cancer-cell channel was measured using a pressure gauge (700G04, Fluke, USA) connected to the channel inlet via a 3-way stopcock.

We used COMSOL Multiphysics 4.4 (COMSOL Inc., USA) to estimate HP profile throughout the device. The inlet and outlet of the simulated T-cell channel were set to be dead ends. The HP applied to the simulated cancer-cell channel was set according to that set in the experiment, namely 1960 Pa (20 cm H_2_O), 980 Pa (10 cm H_2_O), and 490 Pa (5 cm H_2_O). A continuous flow rate of 0.1 μL/min was set in the simulated vessel channel. The medium was set as incompressible fluid with negligible inertia term. To improve the accuracy of numerical simulation, the meshes were set as free tetrahedrons with two different minimum element sizes, which were 0.1 μm and 2 μm within and outside the slit channels, respectively.

## Supplementary information

Supplementary Information.

Supplementary Figure.

Supplementary Video 1.

Supplementary Video 2.

Supplementary Video 3.
