# Supplementary material for: Evaluation of cytotoxic T lymphocyte-mediated anticancer response against tumor interstitium-simulating physical barriers
Source: Sci Rep. 2020 Aug 12;10:13662. doi: 10.1038/s41598-020-70694-8 (PMC7423901; doi:10.1038/s41598-020-70694-8)
Supplement: Supplementary file 1 — Supplementary Information. [file 41598_2020_70694_MOESM1_ESM.pdf]

Evaluation of cytotoxic T lymphocyte-mediated anticancer response against tumor interstitium-simulating physical barriers

Shu-Ching Chen[1#], Po-Cheng Wu[2#], Chiao-Yi Wang[2], and Po-Ling Kuo*[234]

[1] Department of Medical Research, National Taiwan University Hospital, Taipei, Taiwan, 10002.
[2] Graduate Institute of Biomedical Electronics and Bioinformatics, National Taiwan University, Taipei, Taiwan, 10617.
[3] Department of Electrical Engineering, National Taiwan University, Taipei, Taiwan, 10617.
[4] Department of Rehabilitation, National Taiwan University Hospital, Taipei, Taiwan, 10002.

[#]These two authors contributed equally to the manuscript.
Correspondence and requests for materials should be addressed to
P.-L. Kuo, email: poling@ntu.edu.tw

Supplementary information

Figure S1| Photographs of anti-GP33+ HEPA1-6 cancer cells response mediated by the GP33-antigen specific P14 CTLs.

Movie M1| BNL cells killed by a 2C cell.

Movie M2| Anti-GP33+ HEPA1-6 cancer cells response mediated by GP33-antigen specific P14 CTLs.

Movie M3| CTL failing to transmigrate exhibits a “lollipop”-like shape.
