# Supplementary figures and images for: Evaluation of cytotoxic T lymphocyte-mediated anticancer response against tumor interstitium-simulating physical barriers
Source: Sci Rep. 2020 Aug 12;10:13662. doi: 10.1038/s41598-020-70694-8 (PMC7423901; doi:10.1038/s41598-020-70694-8)

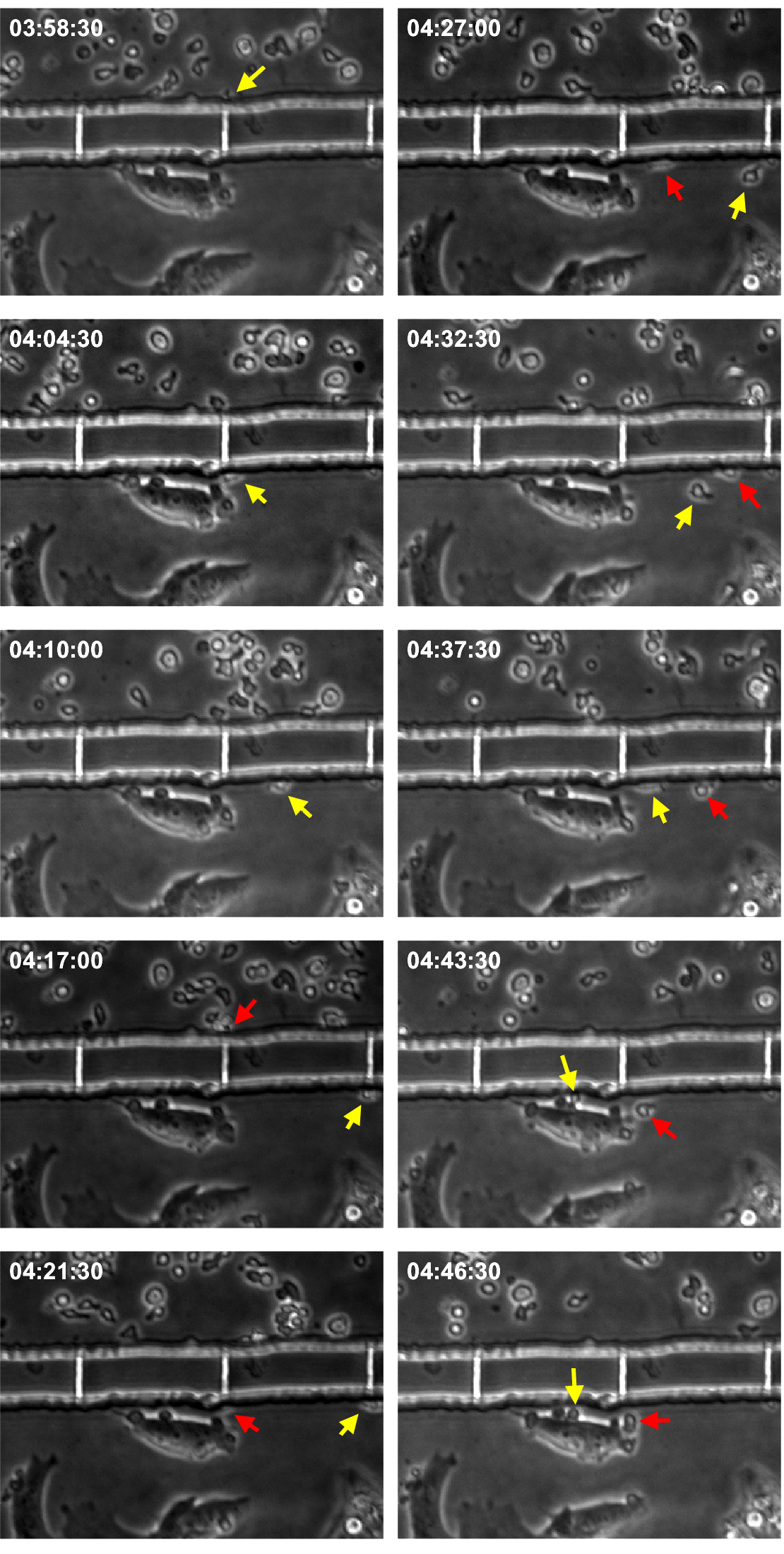

Supplement: Supplementary file 2 — Supplementary Figure. [file 41598_2020_70694_MOESM2_ESM.tif]
